# Population-Based Teacher-Rated Assessment of Anxiety Among Canadian Kindergarten Children

**DOI:** 10.1007/s10578-022-01332-9

**Published:** 2022-03-04

**Authors:** Magdalena Janus, Julia Ryan, Molly Pottruff, Caroline Reid-Westoby, Marni Brownell, Teresa Bennett, Catherine S. Birken, Eric Duku, Mark A. Ferro, Barry Forer, Stelios Georgiades, Jan Willem Gorter, Martin Guhn, Jonathon Maguire, Heather Manson, Jacqueline Pei, Rob Santos, Robert J. Coplan

**Affiliations:** 1 Offord Centre for Child Studies, Department of Psychiatry and Behavioural Neuroscience, McMaster University, BAHT 132, 1280 Main St. West, Hamilton, ON L8S 4K1 Canada; 2 Department of Psychology, Carleton University, Ottawa, ON Canada; 3 Department of Community Health Sciences, Manitoba Centre for Health Policy, University of Manitoba, Winnipeg, MB Canada; 4 Department of Pediatrics, University of Toronto, Toronto, ON Canada; 5 The Hospital for Sick Children, Toronto, ON Canada; 6 School of Public Health and Health Systems, University of Waterloo, Waterloo, ON Canada; 7 Human Early Learning Partnership, School of Population and Public Health, University of British Columbia, Vancouver, BC Canada; 8 Department of Pediatrics, McMaster University, Hamilton, ON Canada; 9 CanChild Centre for Childhood Disability Research, McMaster University, Hamilton, ON Canada; 10 Li Ka Shing Knowledge Institute, St. Michael’s Hospital, Toronto, ON Canada; 11 Public Health Ontario, Toronto, ON Canada; 12 Department of Educational Psychology, University of Alberta, Edmonton, AB Canada

**Keywords:** Kindergarten, Anxiety, Early Development Instrument, Canada, Early child development

## Abstract

Despite anxiety being a prevalent mental health problem in children, little data exist on the pervasiveness and levels of anxiety symptoms in kindergarteners. Data from the *Early Development Instrument*, a teacher-completed, population-level measure of child development, were collected across Canada from 2004 to 2015. The final analytic sample consisted of 974,319 children of whom 2.6% were classified as “highly anxious”. Compared to children who exhibited “few to none” anxious behaviors, highly anxious children were more likely to be male, have English/French as a second language, and have a special needs designation. Furthermore, compared with their less anxious peers, highly anxious children had between 3.5 and 6.1 higher odds of scoring below the 10^th^ percentile cut-off in physical, social, language/cognitive and communication domains. Our findings suggest that anxious behaviors are related to children’s overall health and illustrate the consistency and extensiveness of anxiety at a very young age among Canadian children.

**Supplementary Information:**

The online version contains supplementary material available at 10.1007/s10578-022-01332-9.

## Introduction

Anxiety disorders are widely acknowledged to be among the most common psychological disorders for children and youth; they emerge and can be diagnosed as early as preschool age [1, 2]. Notwithstanding, information regarding the prevalence of anxiety disorders during the early childhood years is quite scarce compared to older children [2, 3]. Lifetime prevalence rates for anxiety disorders in youth seem to span up to 22% [4, 5], and at any given time, nearly 2–3% of children and youth meet criteria for an anxiety disorder [6, 7]. To date, the prevalence research in early childhood typically encompasses preschool children who meet complete diagnostic criteria for an anxiety disorder. For instance, studies have shown prevalence rates of various anxiety disorders among 2–5-year-old children ranging from 0.3 to 11% [3, 8, 9]. These studies typically involve non-psychiatric, community samples, and employ measures such as the *Child Behavior Checklist* (CBCL) [10], the *Preschool Age Psychiatric Assessment* (PAPA) [11], and clinical interviews [3]. For instance, Egger and Angold [3] completed a review of four prevalence studies from 1997 to 2006, all of which utilized non-psychiatric samples and a two-stage approach including the above mentioned questionnaires to identify high scorers followed by clinical interviews. These studies reported a range of specific anxiety disorder prevalence between 0.3 and 5% [3]. In 2015, a population-based study was conducted in Germany recruiting families at school entry (*n* = 1564). Parents of preschool children completed a questionnaire of select items from the CBCL [10] and the DISYPS-II [12], and the prevalence of anxiety disorders ranged from 3.4 to 10.7% [13]. In 2021, a study in Norway recruited a large sample of parents of 4-year-olds (*n* = 3456) during routine check-ups [14]. Parents completed the PAPA [11] and prevalence rates between 0.12 and 0.32% for specific anxiety disorders were reported. Evidently, despite variation in reported prevalence rates across studies, anxiety is common among young children.

Moreover, anxiety in early childhood has consistently been linked to continued experiences of anxiety throughout childhood, adolescence, and adulthood [15–19], as well as to lower quality of life [20]. Importantly, researchers have shown that the occurrence of heightened but *subclinical* levels of anxiety actually exceeds that of formal anxiety disorders, while being equally impairing. For instance, Bell-Dolan, Last and Strauss [21] found that symptoms of anxiety were present in up to 20–30% of non-clinical youths, and other studies have placed estimates ranging between 3% and 49% [21, 22]. Although there is a good deal of variation in these reported rates of anxiety symptoms, these findings clearly indicate that feeling anxious is a very common phenomenon among children and youth.

The substantive impact of subclinical anxiety in childhood, or non-diagnosed but maladaptive anxious emotion, is also evident. There is growing evidence to suggest that heightened symptoms of anxiety put children at risk for future anxiety and mood difficulties [23–27], and are associated with increased substance use in adolescence [28] and poor academic outcomes [25, 29]. Perhaps most consistently, anxiety symptoms appear to be detrimental to children’s academic achievement [24–26, 29, 30]. Indeed, these negative trajectories for children with subclinical but elevated symptoms of anxiety parallel those for children with anxiety disorders [28]. The presence of anxiety among kindergarten age children appears to be of particular importance. Indeed, many researchers have focused attention on the developmental characteristics and qualities that children possess when they enter school, and how these attributes (sometimes referred to as *school readiness*) [31] impact their academic trajectories. Aspects of developmental health, such as academic, cognitive, and socio-emotional skills, are suggested to be necessary for the successful adaptation to school settings, such as the ability to learn, interact with peers and teachers, and follow classroom routines [31–33]. There has been a growing interest in the socioemotional contributors to children’s development at school entry. For instance, Romano et al. [34] reported that anxiety and depression among Canadian kindergarten children were among several factors that significantly predicted math and reading levels in third grade. These researchers concluded that socioemotional factors in kindergarten acted as predictors for later school success. Similarly, Duchesne et al. [24] found that a group of kindergarteners with high levels of anxiety were at greater risk of high school non-completion, even when controlling for other significant risk factors like aggression, hyperactivity, academic achievement, and familial adversity, indicating that socioemotional functioning in kindergarten can be predictive of much later academic outcomes.

There are several well-studied tools available to assess anxiety among older children (i.e., 8 years of age and older). These include the *Multidimensional Anxiety Scale for Children* [35], the *Screen for Child Anxiety and Related Emotional Disorders* [36], and the *Spence Children’s Anxiety Scale* [37]. These are questionnaire-based measures that ask questions specific to symptoms of anxiety, and can be completed by the child themselves, as well as by parents or teachers. For younger children, there are fewer well-established options to choose from. These include the parent-reported *Preschool Anxiety Scale* [38] and relevant subscales from the *Child Behavior Checklist* [39], with both teacher and parent forms. Parent and teacher ratings have generally been found to have good agreement [40]. Unlike parents, however, teachers can report on behaviors observed in school, an environment that might cause some children to be anxious [41], making them a valuable source of information for anxiety in kindergarten children. The current study employed the *Early Development Instrument* (EDI) [31], a teacher-completed checklist that assesses several domains of developmental health including emotional maturity and encompasses anxiety.

The EDI was developed in the late 1990s, in consultation with educators, as a holistic, population-based measure of early child development [26]. Many studies have examined the psychometric properties of the EDI over the years, including construct validity [31, 42, 43], predictive validity [32, 44–46], between-group validity [47, 48], and cross-cultural validity [49–52]. All these studies have consistently demonstrated the validity and reliability of the EDI as a measure of early child development. The EDI includes an “anxious/fearful behaviour” subdomain targeting internalizing problems with mostly anxiety items that are very similar to those of other anxiety measures. Comparison of the prevalence obtained using the EDI resembles those from other studies [53], making it a good choice for evaluating mean levels of anxiety symptoms among kindergarten children. The added advantage of using the EDI in Canada is that data are collected at the population level, that is, for all children who attend publicly-funded kindergarten in a given year. Given the limited evidence on early symptoms of anxiety, the population-level coverage of the EDI, combined with methodological rigor, presents a unique opportunity to conduct a broad and extensive assessment of anxiety symptoms among young children.

### The Current Study

To date, studies reporting prevalence rates of anxiety disorders in youth have varied tremendously. Some studies from around the world (US and Germany) reporting prevalence rates of anxiety in early childhood place estimates around 20% [13, 54], while Canadian figures seem to differ, with about 5% of youth being diagnosed with an anxiety disorder [55]. Using an early subset of the EDI data collected between 2005 and 2007 for just over 170,000 children, Janus [53] found that about 2–3% of Canadian children in the 4–6-year-old range had elevated symptoms of anxiety. However, this study was a preliminary exploration of anxiety symptoms and data came from only 7 out of 13 provinces/jurisdictions thus reflecting a smaller and less representative Canadian sample than the current study. To date, population-level studies of anxiety symptoms in young children remain scarce. Moreover, we know even less about basic questions, such as how anxiety in early childhood is related to demographic variables and other indices of developmental vulnerability. Accordingly, the primary objectives of this study were to: (1) determine teacher-reported mean levels of anxiety symptoms in kindergarten children attending publicly-funded schools across Canadian provinces and territories and their demographics; and (2) examine the associated developmental vulnerabilities among children who exhibit anxious symptoms in this Canadian population sample. Although the EDI does not provide clinical diagnoses of anxiety disorders, we speculated that the established cut off rate that identifies children as *vulnerable* in this domain (i.e., “highly-anxious”—see Method section for more details) would be consistent with previously established prevalence rates for anxiety in early childhood in Canada. Accordingly, it was hypothesized that about 2–5% of kindergarten children in Canada would meet the classification criteria for being *highly anxious*, with some variation across provinces (as a function of underlying differences in population demographics). In addition, based on the aforementioned research suggesting the impairment of anxiety extends to other emotional and academic domains, we also hypothesized that increased ratings of anxiety would be related to increased difficulties in the other developmental domains of the EDI, suggesting broad implications for developmental health. These findings will contribute to the developing body of evidence on anxiety among young children with a focus on a Canadian sample and potentially used for development of preventive strategies.

## Method

### Study Population

The study population includes all children attending publicly-funded schools whose teachers completed the EDI between 2004 and 2015. Teachers completed the EDI once for all the children enrolled in their class at the time of data collection. All EDI data collections from 2004 to 2015 include developmental outcomes along with demographic information for the children. The total study population consisted of 1,038,354 children for whom EDI records were collected in 12 of the 13 provinces and territories in Canada (with the exception of Nunavut). The study received approval from the Hamilton Integrated Research Ethics Board (#2403).

Kindergarten in Canada is the school year before entry to Grade 1, and it is the grade children start the year they turn 5 years old. The vast majority of Canadian children attend publicly-funded schools (91.8% in 2018/19) [56]. Data were collected in the second half of the school year. Data collection occurred in each province/territory^1^ between 1 and 5 times in either a single year or in 2–5-year waves, where a subset of the population was sampled each year until full provincial coverage was achieved (see Table 1). In addition to the ratings on child development, the database includes the following demographic information for each child: age (at time of data collection), sex (male/female), whether they have English/French as a Second Language (E/FSL; yes/no), and whether they have been identified with a special health need (SN; yes/no).

**Table 1:** Canadian EDI implementation schedule from 2004 to 2015 with number of children in each implementation

	AB	BC	MB	NB	NL	NT	NS	ON	PEI	QC	SK	Y
2004								**118,903**				
2005	2449	**35,369**	8387	678						1620	3073	
2006			**11,671**	784			1520			11,186	1280	
2007			**11,514**	383				**115,952**		1394	1538	
2008	191	**37,213**	1418		340		456		**1090**	876	1580	
2009	**72,681**		**11,516**	**6996**			4833			2519	**22,320**	
2010		**46,243**					746	**124,743**				**336**
2011			**11,819**		1088		2295					**340**
2012		**42,033**			2089	**581**	2197			**56,989**	538	**362**
2013			**12,873**		**4833**	**606**	**8394**				7943	**399**
2014		**1277**			**5060**	**606**	1375					
2015			**13,196**			**595**	**8504**	**132,719**				

Bold font in cells indicates a full provincial collection; if the cell spans multiple years it means a province or territory completed the implementation in waves. Regular font in cells indicates a partial provincial collection

*AB* Alberta, *BC* British Columbia, *MB* Manitoba, *NB* New Brunswick, *NL* Newfoundland and Labrador, *NT* Northwest Territories, *NS* Nova Scotia, *ON* Ontario, *PEI* Prince Edward Island, *QC* Quebec, *SK* Saskatchewan, *Y* Yukon

#### Inclusion Criteria

The following inclusion criteria were applied: (1) the child was enrolled in kindergarten; (2) the child was in the classroom for at least one month; and (3) there was no more than 25% of missing items on the child’s EDI. As a result, 47,852 (4.6%) records were excluded from analysis. Of the remaining 990,502 records that were considered valid for analysis, an additional 16,183 (1.6%) records were excluded due to missing data on any of the variables of interest (i.e., province, year of data collection, sex, age, E/FSL, SN, anxiety subdomain, domain vulnerability), resulting in the final analytic sample of 974,319 children. The decision to exclude any cases with missing data was made in order to ensure the same children were included in the unadjusted as well as adjusted regression model. Table 2 summarizes the descriptive statistics of the full study population, those excluded from analyses, and the final analytic sample.

**Table 2:** Descriptive statistics of the full population and final analytic sample

Variable	Full population(N = 990,502)	Excluded from analyses (N = 16,183)	Final analytic sample (N = 974,319)
% Male	51.3	52.0	51.3
Mean age (SD)	5.71 (.32)	5.69 (.34)	5.71 (.32)
% E/FSL	13.0	19.4	12.9
% Special needs	3.6	8.1	3.6
% Anxious	2.6	4.1	2.6

### Measures

The *Early Development Instrument* (EDI) [31] is a population-based, teacher-completed instrument that measures children’s developmental health in five domains: physical health and well-being, social competence, emotional maturity, language and cognitive development, and communication skills and general knowledge. The domains are further broken down into 16 subdomains. The structure of the EDI subdomains within the domains has been confirmed through factor analysis [57]. In particular, the emotional maturity domain contains 30 items divided into the following subdomains: (1) prosocial and helping behavior; (2) anxious and fearful behavior; (3) aggressive behavior; and (4) hyperactivity and inattention. A mean score, from 0 to 10, is calculated for all domains and subdomains, with a higher score indicating greater ability [57]; thus, a high score on the anxious/fearful behavior subdomain means that a child shows few (or none) of those behaviors, while a low score means that they exhibit many of them.

There are two main sets of outcome variables based on the EDI data: the domain mean scores and vulnerability. Vulnerability was used as the dependent variable in the current study. After a jurisdiction collects EDI data at the population-level for the first time, the children’s scores are ranked and then used to create cut-offs. A child is considered to be vulnerable in a given domain if their score falls below the 10th percentile distribution cut-off [57]. Vulnerability on one or more domains has proven to be highly predictive of children’s achievement in later grades (e.g., [46, 58, 59]), as well as meaningful for jurisdictional comparisons in other countries [60].

The independent variable in this study was measured by the anxious and fearful behavior subdomain that is one of four subdomains contributing to the domain of Emotional maturity (8 items: upset when left by parent/guardian; seems to be unhappy, sad, or depressed; appears fearful or anxious; appears worried; cries a lot; nervous, high-strung, or tense; incapable of making decisions; shy; see Appendix 1 for factor loadings) representing symptoms of anxiety*.* The EDI’s Emotional maturity “anxiety and fearful behaviour” subdomain, which represents a broader concept of “internalizing” behaviours, was designed based on items commonly found in established anxiety measures and narrowed down to those that would be most appropriate for children aged between 3.5 and 6.5 years. For example, similar items can be found on relevant scales from the *Child Behavior Checklist* (CBCL, e.g., “cries a lot”, “worries”, “shy or timid”) [61] and the *Strength and Difficulties Questionnaire* (SDQ, e.g., “many worries or often seems worried”, “many fears, easily scared”) [62]. Due to the EDI’s explicit aim to provide a holistic, broad measure of child development, and not be used for clinical assessment, there are no explicit validity studies of the subdomains that would have been required for a diagnostic use of the subdomain scores.

Each of the subdomains has an empirically based threshold [57] that reflects whether the child meets the developmental expectations for the subdomain or not. These thresholds were developed in consultation with educators, early years professionals, and academics working in the field of early childhood development to facilitate the use of the EDI results by stakeholders. Behavioural profiles were developed for school districts based on reviewing the frequencies of item endorsement for children who scored below the 10th percentile vulnerability cut-off for a given domain. This was then subjected to a focus group with kindergarten teachers and an in-person consultation with three child developmental clinicians (one child psychiatrist and two child psychologists). Once agreements were achieved, the score equivalent to the specific endorsement frequencies was calculated. For the anxious/fearful behavior subdomain, this score represents a child who exhibits at least half of the anxious and fearful behaviors “often,” or most of these behaviors (at least 7 out of 8) “sometimes.” Therefore, a child was deemed to meet few or none of the developmental expectations if their mean score was equal to or lower than 4.999 [57]. This threshold was considered to reflect the top of “the lowest range” of developmental skills children should possess in order to present as “ready to learn at school.”

For the purpose of this study, a child was classified as *anxious* if his/her score was below the subdomain threshold. In support of this approach, Janus [53] reported that this method yielded prevalence rates of anxiety comparable with previously established levels in the extant literature. The subdomain was originally derived based on a factor analysis of the emotional maturity domain in the Normative EDI database (*N* = 160,000) [57], with an internal consistency of Cronbach’s α = 0.808. A Confirmatory Factor Analysis (CFA) was performed on the current dataset, with factor loadings reported in Appendix 1. The internal consistency of the subdomain in the present study population was Cronbach’s α = 0.799.

### Analytic Plan

Demographic characteristics and domain vulnerability rates were then examined between anxious and non-anxious children, using chi-square statistics and Cramer’s V to examine effect sizes. In order to examine the association between anxiety and children’s concurrent development in four domains (physical, social, language/cognitive, and communication), unadjusted and adjusted binary logistic regressions were conducted, controlling for sex, age, E/FSL status, SN designation, as well as province and year of data collection. The first four variables are individual child characteristics shown to have strong associations with child development at school entry. Province and year of data collection were added to adjust for any variation in the timing and nature of the data collection initiatives. IBM SPSS Statistics, version 25 [63] was used for all analyses and we employed a level of significance of *p* = 0.004 for our hypothesis testing, using the Bonferroni correction.

## Results

### Prevalence and Demographics

There was a total of 25,262 (2.6%) kindergarten children classified as anxious in Canada. The prevalence of anxiety was significantly different between jurisdictions (*χ*^*2*^(11) = 462.738, *p* < 0.001), ranging from 1.1% in Prince Edward Island (PEI) to 5.0% in Northwest Territories. To investigate whether the prevalence of anxiety was significantly different between individual provinces/territories and the remaining population, province/territory was dummy coded. Although the percentage of anxious children was significantly lower than the national average in four provinces (PEI, Newfoundland, Saskatchewan, Ontario) and significantly higher than the national average in three provinces (Nova Scotia, British Columbia, Northwest Territories), the effect sizes for these differences were all very small (*Cramer’s V* < 0.017, see Table 3). Results from a one-way analysis of variance (ANOVA) determined that the prevalence of anxiety was significantly different across EDI years (ranging from 2.1 to 3.0% per year (*F*(1,974,317) = 238.0, *p* < 0.001), however the effect size was very small (*η*^*2*^ < 0.001) and there was neither an increasing or decreasing pattern over time.

**Table 3:** Chi-square tests (with effect sizes, using Cramer’s V) of the prevalence of anxious children by province/territory

Province	Number (%) of children with anxiety symptoms	Total number of children	χ^2^	Cramer’s V
PEI	12 (1.1)	1075	9.29*	0.003
Newfoundland	213 (1.6)	13,294	52.36*	0.007
Saskatchewan	841 (2.2)	37,915	21.93*	0.005
Ontario	11,660 (2.4)	482,852	120.04*	0.011
Manitoba	2087 (2.6)	80,994	0.09	< 0.001
Quebec	2130 (2.6)	81,506	0.15	< 0.001
Alberta	1910 (2.6)	74,862	0.55	0.001
New Brunswick	233 (2.7)	8651	0.35	0.001
Nova Scotia	871 (2.9)	29,848	12.90*	0.004
British Columbia	5139 (3.2)	159,532	298.38*	0.017
Yukon	48 (3.4)	1423	3.44	0.002
Northwest Territories	118 (5.0)	2367	53.77*	0.007

*p < 0.004, corrected for multiple comparisons

The demographic characteristics of anxious children are presented in Table 4. Compared to their non-anxious peers, anxious children were significantly more likely to be male, have a SN designation, and have E/FSL. Anxious children were also younger than non-anxious children (5.68 vs 5.71 years) as determined by a one-way ANOVA (*F*(1,974,317) = 238.0, *p* < 0.001, *η*^*2*^ < 0.001), however the effect size was very small.

**Table 4:** Demographic characteristics and vulnerability rates in four EDI domains among anxious and non-anxious children

	Anxious %	Non-Anxious %	*χ*^*2*^	Cramer’s V
Demographics				
Male	55.4	51.2	178.1*	0.014
SN	11.1	3.4	4210.0*	0.066
E/FSL	14.2	12.9	37.9*	0.006
Developmental domain vulnerability				
Physical	43.1	11.3	23,446.6*	0.155
Social	42.6	10.3	26,016.3*	0.163
Language-cognitive	27.8	8.8	10,555.8*	0.104
Communication and general knowledge	42.8	13.2	18,029.4*	0.136

*p < 0.004, corrected for multiple comparisons

### Anxiety and Vulnerability in Other Areas of Development

Overall, anxious children were significantly more likely than their non-anxious counterparts to be vulnerable in four domains of the EDI: physical, social, language/cognitive, and communication (Table 4). We then examined the magnitude of this relationship by conducting logistic regression, acknowledging that this was purely an analysis of association as the domains are correlated with each other with coefficients ranging from 0.47 to 0.63, except for the one between emotional maturity and social competence, which was 0.80. Table 5 shows results of the unadjusted and adjusted binary logistic regressions odds ratios, adjusted odds ratios (controlling for children’s sex, age, E/FSL, SN status, province/territory, and year of data collection), as well as corresponding 95% confidence intervals (CIs) for child anxiety and vulnerability on four EDI domains (all but emotional maturity). Anxious children had 3.5 to 6.1 times higher odds of being vulnerable in these domains, compared to their non-anxious counterparts.

**Table 5:** Binary logistic regression analyses examining the associations between child anxiety and vulnerability in the four developmental domains of the EDI

Developmental domain	Unadjusted odds ratio (95% CI)	Adjusted odds ratio (95% CI)^a^
Physical health and well-being	5.96 (5.81–6.12)	5.44 (5.29–5.59)
Social competence	6.48 (6.32–6.65)	6.14 (5.97–6.31)
Language and cognitive development	3.99 (3.88–4.11)	3.46 (3.35–3.56)
Communication and general knowledge	4.94 (4.81–5.06)	4.93 (4.79–5.07)

^a^Controlled for children’s sex, age, E/FSL and SN statuses, province/ territory, and year of EDI data collection

## Discussion

This study provided the first pan-Canadian snapshot of anxiety symptoms among Canadian children at school entry. Prevalence varied somewhat across Canadian jurisdictions, ranging from 1.1% in PEI to 5.0% in Northwest Territories. Our results confirm the hypothesis that the prevalence of anxiety would be comparable to epidemiological sample-based studies of children of comparable age (i.e., 2–3% from [53]).

We also investigated demographic trends in variation of anxiety symptoms, including age, sex, E/FSL, SN status, and region (province/territory). Children identified as *highly anxious* tended to be younger and were more likely to be male than their non-anxious counterparts. However, it should be noted that although both of these differences were statistically significant, the effect sizes were quite small. Since anxiety has been consistently documented in this age range, the age effects here likely represent minor differences in anxiety among a single grade year, rather than a broad age-based effect. In other words, we can use these data to predict that students who are younger than their peers may be more prone to demonstrating anxiety in the classroom for factors related to development and maturity. In terms of sex, anxiety has typically been found to be more prevalent among females than males across the lifespan (e.g. [64–66]), but this effect has not always been found (e.g. [67, 68]), and some suggest it may only emerge in adolescence [69, 70]. Our data suggest that the higher prevalence of anxiety in females may not be present in the early childhood age range, and if anything, indicate a possibility of the reverse pattern.

Anxious children also tended to have SN and E/FSL at a higher rate than their non-anxious counterparts. Indeed, the associations between anxiety and second language learning, as well as between anxiety and SN, have been well documented to date (e.g., [71–75]). For instance, researchers have found higher incidences of anxiety among children with intellectual delays [74, 76] and with autism [77, 78]. Although various factors have been attributed to this phenomenon, emotional problems that co-occur with these disorders seem likely (e.g. [79–81]). With regard to second language learning and anxiety, this phenomenon has been attributed to various possible causes, such as communication apprehension and fear of negative evaluation [82], stressful classroom experiences and perception of language aptitude [83], as well as various interactions between the teacher, the learner, and the classroom [84, 85]. Regardless of the possible explanations, our data provide further evidence that young children with SN or those who are learning in a second language demonstrate higher levels of anxiety in the classroom.

Another notable finding is that prevalence of highly anxious children differed between provinces/ territories, though with only a small effect size. This may be due, at least in part, to provincial/territorial differences around teacher familiarity with these constructs, or systemic differences in the education settings. First, education in Canada is placed within a provincial, rather than a federal jurisdiction, and thus teacher training and curricula vary across the country. In addition, the kindergarten structure also varies widely across Canada, from half day, to full day part-time, to full day, every day. In fact, in our sample, Ontario instituted a change to make full day kindergarten available during the 3rd cohort (representing about a quarter of the total Ontario sample). Indeed, this sort of schedule change could impact teacher ratings of children’s anxious behavior due to teachers spending more time with students and having more opportunities to observe them. Perhaps relatedly, we found a small, but statistically significant effect of cohort (year of study), pointing to the possibility that these types of changes may affect anxiety ratings in students. Together, these results suggest that it may be useful to conduct future studies that focus on cohort effects and region-specific factors that may be at play.

We also examined the links between anxiety and other aspects of developmental health. Our results demonstrate that having symptoms of anxiety is not benign: children with elevated symptoms of anxiety are three to six times more likely to be vulnerable on other domains of development, that is, physical, social, language/cognitive, and communication skills, than those who exhibit very few of these symptoms. This finding supports the notion that anxiety and other aspects of development, as well as skill acquisition and use, are closely intertwined, and further promotes a holistic view of developmental health that includes socio-emotional functioning.

### Strengths and Limitations

This study is an extensive examination of anxiety symptoms among kindergarten students in Canada, a first national investigation in this age group. A large sample was used with a robust method of population-based data collection with developmentally appropriate instrument that enhances representativeness, and that is comparable to other behavioral assessments in this age group. In addition, the data allowed us to examine region-specific prevalence rates of anxiety, which can provide important information for policy. The inclusion of other domains of developmental health in conjunction with anxiety prevalence is also a unique contribution, highlighting the importance of anxiety in the context of developmental health more broadly.

Even though our study population represents a large proportion of children attending publicly-funded kindergarten classes, the children studied here do not represent *all* kindergarten-aged children in Canada. About 92% of school aged children are enrolled in public schools across Canada [86], and so children who attend private school or are home-schooled are not included in the current sample. In addition, the children included here varied in type of kindergarten setting (full day or half day, every day or every other day). The difficulty with addressing program type is that it is confounded by province/territory. Because kindergarten programs are implemented at the provincial level, controlling for province/territory in our analyses also accounted for program type. Previous research suggests that the association between type of care a child receives and child anxiety is a complex and non-linear relation that appears to vary as a function of both quantity and type of care (e.g., [87]). This makes it difficult to ascertain how the results of the current study might have been influenced by full- versus half-day kindergarten experiences, or whether anxiety among children who are home schooled or in private schooling may differ from the children included here. Targeting these questions in future studies would be helpful in determining risk and protector factors in the presentation of anxiety symptoms among kindergarten age children.

The anxiety measure used here has not been explicitly tested for convergent or discriminant validity with diagnostic tools, although it includes items very similar to those used in such tools (e.g., [61, 62]). This study includes a single rater, observer-based assessments of anxiety, limiting the interpretation of this prevalence data. Using observer-based ratings exclude internalizing factors that may not be noticeable to the informant. Similarly, relying on teacher reports excludes anxious behavior that may be occurring at home, as reported by parents. Teacher reports do provide meaningful information about student behavior in school, an important domain of functioning, providing relevance to the examination of these symptoms as displayed and observable in a developmentally-relevant context. Therefore, the data presented here do not provide a comprehensive view of a child’s anxious behaviors and symptoms across different settings, but rather an estimate of anxious behaviors among children in a specific academic setting. While the EDI is not a screening tool, the experience of completing it does alert the teacher to potential difficulties a child may face and thus could be used as a basis for further assessment with clinically validated measures of anxiety to determine the severity of the issue and need for intervention.

## Summary

In this first population-wide study of anxiety symptoms in young Canadian children, we found slight differences across provinces and territories, with a national prevalence average of 2.6%. We also found that rates were fairly stable over the years, with slight variations. Symptoms of anxiety in kindergarten children, as reported by their teachers, were associated with increased odds of being vulnerable in other areas of their development which indicates that children who are anxious are more likely to also struggle in those areas than their non-anxious counterparts. Altogether, the current study demonstrates the feasibility of using the EDI data to monitor anxiety, as displayed at school, in kindergarten children.

Early identification is a crucial step in providing early intervention and prevention programs for young anxious children that attempts to mitigate the long-term impact of anxiety. In recent years, several efficacious programs have been developed targeting young children vulnerable to early anxiety (e.g., high in behavioral inhibition) (see [88] for a recent review). These include parental education and training programs (e.g., Cool Little Kids, [89]) and multi-component interventions that also incorporate social skills training (e.g., Turtle Program, [90]). In jurisdictions that implement the EDI on a regular basis, the frequency of anxiety symptoms on the questionnaire can be used to monitor the impact of interventions employed in preschools. Schools with children with high frequencies of anxiety symptoms may be advised to put into practice class-level activities to mitigate long-term consequences of kindergarten anxiety.

In future, corroboration of these prevalence rates by including parent-ratings would be useful. Similarly, validating the use of the EDI as a measure of anxiety would be a worthwhile next step, such as by including diagnostic interviews to confirm anxiety symptom levels and diagnostic status. Finally, this study provides “baseline” estimates of anxiety symptoms among kindergarten-aged children in Canada, which could, in future, be compared to data collected with the same method among the post-COVID-19 cohorts of kindergarten-age students across Canada.

## Supplementary Information

Below is the link to the electronic supplementary material.

Supplementary file1 (DOCX 17 kb)

## Footnotes

1 Each jurisdiction funds its own population-level data collection and decides the frequency of data collections for their province/territory. The timing of the different collections is therefore out of the authors’ control.
