# Supplementary material for: Population-Based Teacher-Rated Assessment of Anxiety Among Canadian Kindergarten Children
Source: Child Psychiatry Hum Dev. 2022 Mar 4;54(5):1309–20. doi: 10.1007/s10578-022-01332-9 (PMC8894824; doi:10.1007/s10578-022-01332-9)
Supplement: Supplementary file 1 — Supplementary file1 (DOCX 17 kb) [file 10578_2022_1332_MOESM1_ESM.docx]

1. **Appendix**

Table A.1

*Results of a Confirmatory Factory Analysis of the anxious and fearful behavior subdomain of the EDI, including factor loadings (β) for each item*

| Item | Estimate | SE | Z | P(>\|z\|) | β |
| --- | --- | --- | --- | --- | --- |
| is upset when left by parent/guardian | 1.000 |  |  |  | .378 |
| seems to be unhappy, sad, or depressed | 1.437 | 0.004 | 327.65 | <0.001 | .600 |
| appears fearful or anxious | 1.934 | 0.005 | 355.80 | <0.001 | .846 |
| appears worried | 2.022 | 0.006 | 355.19 | <0.001 | .833 |
| cries a lot | 0.999 | 0.003 | 306.41 | <0.001 | .493 |
| is nervous, high-strung, or tense | 1.460 | 0.004 | 336.57 | <0.001 | .665 |
| is incapable of making decisions | 1.231 | 0.004 | 303.34 | <0.001 | .499 |
| is shy | 1.327 | 0.005 | 273.22 | <0.001 | .401 |

Fit statistics and indices: χ^2^(20) = 2361568.804, p < 0.001; RMSEA = 0.090; SRMR = 0.042; CFI = 0.933; TLI = 0.906

Note: RMSEA = Root Mean Square Error of Approximation; SRMR = Standardized Root Mean Residual; CFI = Comparative Fit Index; TLI = Tucker Lewis Index
